# Mannan Oligosaccharides Application: Multipath Restriction From *Aeromonas hydrophila* Infection in the Skin Barrier of Grass Carp (*Ctenopharyngodon idella*)

**DOI:** 10.3389/fimmu.2021.742107

**Published:** 2021-10-18

**Authors:** Zhiyuan Lu, Lin Feng, Wei-Dan Jiang, Pei Wu, Yang Liu, Jun Jiang, Sheng-Yao Kuang, Ling Tang, Shu-Wei Li, Xiang-An Liu, Cheng-Bo Zhong, Xiao-Qiu Zhou

**Affiliations:** ^1^Animal Nutrition Institute, Sichuan Agricultural University, Chengdu, China; ^2^Fish Nutrition and Safety Production University Key Laboratory of Sichuan Province, Sichuan Agricultural University, Chengdu, China; ^3^Key Laboratory for Animal Disease-Resistance Nutrition of China Ministry of Education, Sichuan Agricultural University, Chengdu, China; ^4^Sichuan Animal Science Academy, Sichuan Animtech Feed Co. Ltd, Chengdu, China; ^5^Animal Breeding and Genetics Key Laboratory of Sichuan Province, Animal Nutrition Institute, Sichuan Academy of Animal Science, Chengdu, China

**Keywords:** mannan oligosaccharides, antioxidant, apoptosis, tight junction, skin immune, grass carp (*Ctenopharyngodon idella*)

## Abstract

The objective of this study was to evaluate the efficacy of dietary Mannan oligosaccharides (MOS) supplementation on skin barrier function and the mechanism of on-growing grass carp (*Ctenopharyngodon idella*). Five hundred forty grass carp were fed for 60 days from the growing stage with six different levels of MOS diets (0, 200, 400, 600, 800, and 1,000 mg kg^-1^). At the end of the growth trial, the 14-day *Aeromonas hydrophila* challenge experiment has proceeded. The obtained data indicate that MOS could (1) decline skin lesion morbidity after being challenged by the pathogenic bacteria; (2) maintain physical barrier function *via* improving antioxidant ability, inhibiting excessive apoptosis, and strengthening the tight junction between the epithelial cell and the related signaling pathway (Nrf2/Keap1, p38MAPK, and MLCK); and (3) regulate immune barrier function by modulating the production of antimicrobial compound and expression of involved cytokines and the related signaling pathway (TOR and NFκB). Finally, we concluded that MOS supplementation reinforced the disease resistance and protected the fish skin barrier function from *Aeromonas hydrophila* infection.

## Introduction

Due to large-scale intensive production facilities, fish are exposed to potential various pathogens that often result in massive economic losses (1). The disease resistance of fish mainly depends on the main defense organs’ immune function (2). Skin, an important mucosal defense organ in fish, has developed a better barrier system (including physical barriers and immune barriers) to protect the whole body from natural pathogen invasion (3). A previous report has confirmed that mechanical skin barrier injury could further lead to high morbidity and mortality of fish (4). Therefore, a protected fish skin barrier function must be necessary for fish health. An effective strategy is to supplement the dietary with prebiotics. The current definition of prebiotics is, “Prebiotics are food constituents that well thought-out to be non-digestible selectively fermented, confers benefits of growth and activity of beneficial microbes present in gastrointestinal tract and improve the health of host” (5). In general, the prebiotics used in animal production are some functional oligosaccharides (6). Studies on fish reported that skin physical and immune barrier function could be improved by functional oligosaccharides such as xylooligosaccharides (XOS), galactooligosaccharides (GOS), fructooligosaccharides (FOS), and so on (7–9). Mannan oligosaccharide (MOS), a kind of functional oligosaccharide, is widely used in the feed formulation of aquatic (10). However, no systematic research has been conducted, and no in-depth exploration has been performed about the relationship between MOS and skin barrier function. Limited research on skin has shown that MOS supplementation promoted mucus production in rainbow trout (*Oncorhynchus mykiss*) and European sea bass (*Dicentrarchus labrax*), and upregulated *IFNγ* and *IL-10* expression in greater amberjack (*Seriola dumerili Risso* 1810) (11–13). Thus, a comprehensive understanding of the effect of MOS on fish skin barrier function and the in-depth possible mechanisms is necessary.

Previous studies have reported that cellular structure and intercellular junctions comprise skin physical barrier, which is mainly related to the antioxidant capacity, apoptosis levels, and tight junctions (1, 14). As far as we know, the NF-E2-related factor 2 (Nrf2) could eliminate excess free radicals by regulating the levels of antioxidant enzymes, while p38 mitogen-activated protein kinase (MAPK) could dynamically regulate apoptosis by regulating apoptosis promoter and effector, thus corporately protecting the cellular structure integrity (15, 16). Myosin light chain kinase (MLCK) is an important signaling molecule that could maintain intercellular junctions by regulating the expression of downstream tight junction protein molecules (17, 18). However, the research to date about the effects of MOS on fish skin cellular structure and intercellular junctions and their possible mechanism has not been investigated. It is worth noting that available evidence suggests a probable correlation between MOS and skin physical barrier. A study on chicken macrophages demonstrated that MOS could increase the production of nitric oxide (NO) (19), which could activate Nrf2 in PC12 cells (20). Furthermore, MOS supplementation could improve calcium (Ca^2+^) absorption and retention in layer hens (21). Other reports revealed that Ca^2+^ induced apoptosis *via* activating p38MAPK signaling pathways in murine macrophage cells (22). Besides, IL-1β gene expression was upregulated by MOS in European sea bass (23). And occludin expression could be decreased by IL-1β in Caco-2 cells (24). These intriguing observations implicate a probably delicate link between MOS and fish skin physical barrier, and the underlying mechanism warrants further exploration.

Fish physical barrier function of the skin is also associated with the immune barrier function, which is closely related to antimicrobial compounds [such as lysozyme (LZ), complement 3 (C3), and immunoglobulins (Ig)] and inflammatory cytokines (25–27). However, available literature describing the skin barrier’s function affected by MOS supplementation after pathogen infections is particularly scarce. A study in human macrophages showed that cytokines were mediated by nuclear factor kappa B (NFκB) (28) and the target of rapamycin (TOR) signaling pathways (29). It has been reported that MOS increased the digestibility of protein in the ileum of piglets (30). Our lab’s previous work in grass carp confirmed that protein increased the activity of LZ and the concentration of C3 (31). A study on weaned piglets demonstrated that fed MOS diet could enhance the digestibility of phosphorus in ileum (30). Another study from our lab in grass carp described that phosphorus could upregulate interleukin 15 (IL-15) expression, which is regulated by the TOR signaling pathway (32). Furthermore, Pinheiro et al. (33) demonstrated that MOS increased butyrate concentration in growing rabbit cecum. It was of note that butyrate could inhibit the activation of the NFκB signaling pathway in grass carp (34). All of these studies imply that MOS might regulate skin immune barrier function *via* acting on multiple pathways, the mechanism of which is worth in-depth exploration.

Based on the lab’s previous MOS study of growth and intestinal health (35), the objectives of the present study were to elaborate on the protective effects of dietary MOS supplementation on the skin barrier function of on-growing grass carp under the condition of pathogen infection. For this purpose, this work explores the influence of MOS on antioxidant parameters, apoptosis parameters, tight junction (TJ) proteins, antibacterial compounds, and cytokines, as well as the possible signal molecule Nrf2, p38MAPK, MLCK, NFκB, and TOR in the skin of grass carp after being challenged with *Aeromonas hydrophila* for the first time. Furthermore, as we all know, the grass carp is a broadly distributed species over the world (36). These results will shed new light on the understanding of freshwater fish defense mechanisms to bacterial pathogens, and also provide a more effective alternative reference for antibiotics.

## Materials and Methods

### Study Design

The method of MOS (Sciphar Hi-Tech Industry, Xi’an, purity: 99.12%) diet preparation and storage was based on our published work (35, 37). The experimental diet formulation and proximate composition analyses are displayed in **Supplementary Table 1**. The different levels of MOS (0, 200, 400, 600, 800, and 1,000 mg kg^-1^) were added to the control diet in place of cornstarch. All completed diets were stored at 4°C until feeding.

### Determination of Antioxidant Properties

MOS antioxidant properties were determined mainly by the kit list in **Supplementary Table 2**. In short, DPPH, ·O^2^ (ASA), and ·OH (AHR) radical scavenging activities of MOS at different levels were determined to reflect the antioxidant properties of MOS *in vitro*. The method used is spectrophotometry as previously described (38, 39).

### Animals and Experimental Management

The guidelines for the Laboratory Animals Care and Use of Animal Nutrition Institute (LACUANI), Sichuan Agricultural University were strictly followed (permit no. LZY-2018114005) during the whole feeding trial. All healthy on-growing grass carp were obtained from Tong Wei fisheries (Sichuan, China) and acclimated to the fishpond culture condition for a month before the experiment. A total of 540 individuals (215.85 ± 0.30 g) were randomized to 18 nylon cages (n=30), and the feeding frequency and experimental period had the same description as our previous study (35). Routine test control parameters were as follows: dissolved oxygen > 6.0 mg L^-1^, water temperature at 28.5 ± 2.0°C, pH value 7.5 ± 0.3, and experiment condition with a natural light cycle during the whole experimental period.

### Challenge Test

After the growth trial, a 14-day challenge test (CT) was conducted to study the effect of dietary MOS on the fish skin barrier function according to our published work (35). Briefly, randomly selected five fish per replicates from each MOS group were intraperitoneally injected with 1.0 ml *A. hydrophila* (FDL20120711), and the concentration of bacteria is 2.5 × 10^8^ colony-forming units (CFU) ml^-1^. Concurrently, the saline group was injected with the same amount of normal saline. The situation and management were in line with the feeding trial. In a previous study, we successfully establish the *A. hydrophila* challenge model.

### Sampling and Biochemical Parameter Analysis

At the end of the CT, all grass carp were anesthetized in a benzocaine bath according to LACUANI requirements. Then fish skin was rapidly collected and temporarily stored in liquid nitrogen. Finally, the sample was stored at -80°C for later analysis. The methods of the skin lesion morbidity scoring system were from a previous study (40). For the determination of physical and immune barrier-related parameters, 10% (w/v) of skin tissue homogenates were prepared with saline (4°C) and centrifuged (6,000 g, 20 min). Then the supernatant was collected. The biomarkers and related enzyme activity analysis methods are shown in **Supplementary Table 2**.

### DNA Fragment Analysis

The fragmented DNA of the skin tissue was isolated as previously described (41). And then, DNA was extracted following the instructions and analyzed on a 2% agarose gel to verify DNA fragmentation. Electrophoresis duration and related parameters were 90 min and 80 V, respectively. Finally, Gene Genius (Syngene, Frederick, MD, USA) is used to analyze the results of visualizations.

### Real-Time PCR

*q*RT-PCR was conducted to refer to the method from our previous work (35). In short, the total RNA of skin samples was isolated by using an RNAiso Plus Kit (Takara, Dalian, China). RNA quality was assessed by 1% agarose gel electrophoresis and quantified by spectrophotometry at 260/280 nm using Nanodrop 2000 (Thermo Scientific, USA). Afterward, RNA was reverse-transcribed into cDNA by using a PrimeScript™ RT reagent kit (Takara, Dalian, China). For *q*RT-PCR, specific primers were designed according to the sequences we cloned (**Supplementary Table 3**). Our preliminary experiment screened four internal reference genes and finally selected *β-Actin* and *GAPDH* as previously described (35, 42). Preparation of melting curves and calculation of amplification efficiency of target genes were according to the manufacturer’s instruction. The gene transcription level was calculated as described by the method (2^−ΔΔCT^) from Livak and Schmittgen (43).

### Western Blot Analysis

Preparation method-related parameters of skin homogenates, primary and second antibodies, and blotting analysis were performed as our lab previously described (35, 44, 45). Extraction and determination of tissue protein were performed by using the RIPA and BCA assay kit (Beyotime). The prepared sample (40 μg lane^-1^) was separated by SDS-PAGE (10%) and transferred to a PVDF membrane. Membranes were incubated overnight with primary antibody (14 h, 4°C). Afterward, membranes were washed and secondary antibody was incubated (90 min, room temperature). Then, protein signals were visualized and quantified (NIH Image J, 1.42q) as previously described (35, 37). All antibodies’ detailed information in the current study is listed in **Supplementary Table 4**.

### Statistical Analysis Method

Before statistical analysis, the Shapiro–Wilk test of normality, as well as Levene’s test of variance homogeneity, was conducted. All data underwent one-way analysis of variance (ANOVA) followed by Duncan’s multiple comparisons at *P* < 0.05 with SPSS 25.0 (SPSS Inc., Chicago, IL, USA). Data visualization was done using the GraphPad 8.0 software (GraphPad Software, Inc.), R (v4.0.2), and Hiplot platform (https://hiplot.com.com).

## Results

### Skin Morbidity and Phenotype

**Figure 1:**
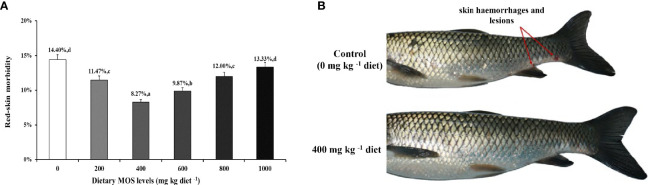
MOS alleviates skin damage of on-growing grass carp after infection of *Aeromonas hydrophila*. **(A)** The red-skin morbidity of fish. Data were represented as the mean ± SD. N = 15 for each MOS level; different letters above bars indicate significant differences (*P* < 0.05). **(B)** The apparent symptoms (red arrow, hemorrhages, and lesions) of fish skin.

To investigate the effect of MOS on fish skin morbidity and phenotype with *A. hydrophila* challenge, we performed intraperitoneal injection of bacteria solution. We obtained the results of skin morbidity and phenotype as showed in **Figure 1**; compared to the control (14.40%), the morbidity of skin hemorrhages and lesions after being challenged was significantly decreased with MOS at 400 mg kg^-1^ diet. At this optimal MOS supplementation, the skin morbidity was reduced to a minimum 8.27% (*P* < 0.05). Then, it showed an upward trend (from 9.87% to 13.33%, *P* < 0.05) with the increase in MOS (600-1000 mg kg^-1^). These results suggest that the optimal level of MOS could effectively reduce skin morbidity.

### Antioxidant Properties of MOS

To investigate whether MOS have antioxidant properties *in vitro*, we designed MOS with different concentrations to test their antioxidant properties (**Supplementary Figure 1**). Our results showed that the free radical scavenging rate (DPPH, ASA, and AHR) increased gradually (from 0 to 60%) with the increase in the dosage of MOS (from 0 to 5 mg ml^-1^) in a dose-dependent manner. These data suggest that MOS has excellent antioxidant properties.

### Biochemical Analysis Parameters

**Figure 2:**
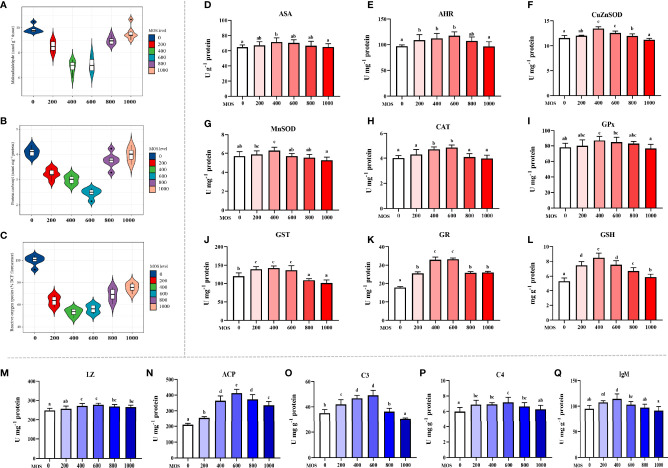
Effect of dietary MOS supplementation on barrier function in the skin of on-growing grass carp after infection of *Aeromonas hydrophila*. **(A–C)** Biomarkers of oxidative damage; ROS, reactive oxygen species (% DCF florescence); MDA, malondialdehyde (nmol g^-1^ tissue); PC, protein carbonyl (nmol mg^-1^ protein). **(D–L)** Antioxidant-related parameters; ASA, anti-superoxide anion (U g^-1^ protein); AHR, anti-hydroxy radical (U mg^-1^ protein); CuZnSOD, copper/zinc superoxide dismutase (U mg^-1^ protein); MnSOD, manganese superoxide dismutase (U mg^-1^ protein); CAT, catalase (U mg^-1^ protein); GPx, glutathione peroxidase (U mg^-1^ protein); GST, glutathione reductase (U mg^-1^ protein); GR, glutathione reductase (U mg^-1^ protein); GSH, glutathione (mg g^-1^ protein). **(M–Q)** Immune-related parameters; LZ, Lysozyme activity (U mg ^-1^ protein); ACP, acid phosphatase (U mg^-1^ protein); C3, complement 3 (mg g^-1^ protein); C4, complement 4 (mg g^-1^ protein); IgM, immunoglobulin M (mg g^-1^ protein). N = 6 for each MOS level; different letters above bars indicate significant differences (*P* < 0.05).

To uncover the MOS effect on fish skin antioxidant capacity with *A. hydrophila* challenge, we determined the content of oxidative damage biomarkers and the activity of key antioxidant enzymes (**Figures 2A–L**). Oxidative damage biomarkers are indicators that reflect the state of oxidative damage. In **Figures 2A–E**, compared with the control diet (ROS: 100% DCF florescence; MDA: 9.88 nmol g^-1^ tissue; ASA: 64.71 U g^-1^ protein), the ROS and MDA contents were decreased, the ASA were increased with the MOS supplementation, and ROS and MDA reached their minimum value (ROS: 53.20% DCF florescence; MDA: 6.88 nmol g^-1^ tissue, *P* < 0.05), whereas ASA reached its maximum value (ASA: 71.46 U g^-1^ protein, *P* < 0.05) with 400 mg kg^-1^ MOS supplementation. Then ROS and MDA showed an upward trend (ROS: from 56.08 to 75.50% DCF florescence, *P* < 0.05; MDA: from 7.07 to 9.60 nmol g^-1^ tissue), and ASA showed a downward trend (ASA: from 70.28 to 64.94 U g^-1^ protein) with the increase in MOS (600–1,000 mg kg^-1^). The PC contents were significantly decreased, and the AHR were increased with MOS supplementation with MOS at 600 mg kg^-1^ diet; at this optimal MOS supplementation, the PC content was obviously reduced to a minimum 2.47 nmol mg^-1^ protein, and AHR was increased to a maximum 117.4 U mg^-1^ protein (*P* < 0.05) compared with the control group (PC: 4.10 nmol mg^-1^ protein; AHR: 97.12 U mg^-1^ protein). Then PC showed an upward trend (from 3.76 to 3.98 nmol mg^-1^ protein), and AHR showed a downward trend (from 66.52 to 64.94 U mg^-1^ protein) with the increase in MOS (800–1,000 mg kg^-1^). These data suggest that the MOS could effectively alleviate oxidative damage caused by *A. hydrophila*.

Antioxidant enzymes are key proteins that scavenge free radicals, and their activities reflect antioxidant capacity. **Figures 2F–L** presents the results obtained from the biochemical analysis of the antioxidant enzymes and non-enzymatic antioxidants (GSH). Compared with the control diet (CuZnSOD: 5.85 U mg^-1^ protein; MnSOD: 5.70 U mg^-1^ protein; GPx: 78.00 U g^-1^ protein; GST: 120.54 U mg^-1^ protein, GSH: 5.28 mg g^-1^ protein), the activity of CuZnSOD, MnSOD, GPx, and GST and the content of GSH were increased with the MOS supplementation, and all of them reached their maximum value (CuZnSOD: 7.18 U mg^-1^ protein; MnSOD: 6.28 U mg^-1^ protein; GPx: 87.11 U g^-1^ protein; GST: 142.34 U mg^-1^ protein, GSH: 8.49 mg g^-1^ protein, *P*<0.05) with 400 mg kg^-1^ MOS supplementation. Then all of them showed a downward trend (CuZnSOD: from 6.84 to 5.94 U mg^-1^ protein; MnSOD: from 5.71 to 5.26 U mg^-1^ protein; GPx: from 84.77 to 76.73 U mg^-1^ protein; GST: from 136.80 to 101.19 U mg^-1^ protein; GSH: from 7.55 to 5.88 mg g^-1^ protein) with the increase in MOS (600–1,000 mg kg^-1^). The activity of CAT and GR increased with the MOS supplementation, and both of them reached their maximum value (CAT: 4.85 U mg^-1^ protein; GR: 33.23 U mg^-1^ protein, *P* < 0.05) with 600 mg kg^-1^ MOS supplementation compared with the control group (CAT: 4.01 U mg^-1^ protein; GR: 17.79 U mg^-1^ protein). Then both of them showed a downward trend (CAT: from 4.09 to 3.98 U mg^-1^ protein; GR: from 25.90 to 26.12 U mg^-1^ protein) with the increase in MOS (800–1,000 mg kg^-1^).

The antimicrobial compound-related parameters are displayed in **Figures 2M–Q**. Compared with the control (LZ: 248.64 U mg^-1^ protein; ACP: 209.33 U mg^-1^ protein; C3: 35.01 mg g^-1^ protein; C4: 5.97 mg g^-1^ protein), the activity of LZ and ACP and the contents of C3 and C4 were increased with the MOS supplementation. And all of them reached their maximum value (LZ: 277.72 U mg^-1^ protein; ACP: 412.41 U mg^-1^ protein; C3: 49.19 mg g^-1^ protein; C4: 7.17 mg g^-1^ protein, *P* < 0.05) with 600 mg kg^-1^ MOS supplementation. Then all of them showed a downward trend (LZ: from 269.13 to 265.80 U mg^-1^ protein; ACP: from 373.30 to 335.22 U mg^-1^ protein; C3: from 36.29 to 30.57 mg g^-1^ protein; C4: from 6.65 to 6.28 mg g^-1^ protein) with the increase in MOS (800–1,000 mg kg^-1^). The IgM content was significantly increased with MOS supplementation with MOS at 400 mg kg^-1^ diet; at this optimal MOS supplementation, the IgM content was obviously increased to a maximum 114.49 mg g^-1^ protein (*P* < 0.05) compared with the control group (IgM: 94.90 mg g^-1^ protein). Then it showed a downward trend (IgM: from 103.00 to 91.59 mg g^-1^ protein) with the increase in MOS (600–1,000 mg kg^-1^).

### Skin Physic Barrier Function Gene Expression

**Figure 3:**
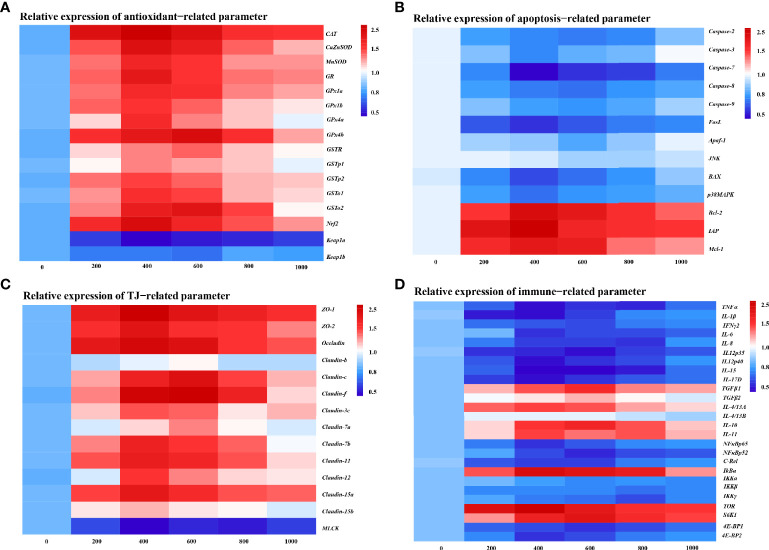
Heat map of MOS (mg kg^−1^ diet) changed expression of antioxidant **(A)**, apoptosis **(B)**, TJs **(C)**, and immune **(D)** related parameters in the skin of on-growing grass carp after infection of *Aeromonas hydrophila*. The signal values of upregulation (red) and downregulation (blue) were expressed and ranged from 0.5 to 2.5 folds.

To further determine the MOS effect on fish skin physic barrier function with *A. hydrophila* challenge, the mRNA expression of the antioxidant, apoptosis, and tight junction-related gene was examined by real-time RT-PCR (**Figures 3A-C**). The enzymatic antioxidant pathway is an important part of the antioxidant system in fish (46). **Figure 3A** provides the heat map of the antioxidant-related gene expression. In comparison with the control group, almost all antioxidant enzyme-related isoforms, *CuZnSOD* (1.84-fold change), *MnSOD* (1.70-fold change), *CAT* (1.98-fold change), *GR* (1.79-fold change), *GPx1a* (1.69-fold change), *GPx1b* (1.63-fold change), *GPx4a* (1.69-fold change), *GSTp1* (1.49-fold change), *GSTp2* (1.61-fold change), and *GSTo1* (1.65-fold change), were significantly upregulated with optimal MOS supplementation up to 400 mg kg^-1^ (*P* < 0.05), and *GPx4b* (1.87-fold change), *GSTo2* (1.81-fold change), and *GSTR* (1.53-fold change) were significantly upregulated with optimal MOS supplementation up to 600 mg kg^-1^ (*P* < 0.05); then all of them followed a gradual downward trend with the increase in MOS (600–1,000 mg kg^-1^ or 800–1,000 mg kg^-1^). Furthermore, the key transcriptional factor *Nrf2* mRNA levels (1.88-fold change) were significantly upregulated with optimal MOS supplementation up to 400 mg kg^-1^ (*P* > 0.05), followed by a gradual downward trend with the increase in MOS (600–1,000 mg kg^-1^). Conversely, the *keap1a* (0.56-fold change) mRNA level had a significant downward trend with 400 mg kg^-1^ MOS supplementation (*P* > 0.05) and then plateaued. However, one of the interesting results we found was that the MOS supplementation did not affect *keap1b* mRNA levels. As expected, our antioxidant gene expression data were consistent with enzyme activities results, suggesting that the optimal level of MOS could enhance the antioxidant capacity of fish skin under *A. hydrophila* challenge.

We investigated the effect of MOS on the apoptosis level by DNA fragmentation and determination of apoptotic pathway gene expression (**Supplementary Figure 2** and **Figure 3B**). **Supplementary Figure 2** provides the visualization results that revealed that skin DNA showed an obvious fragmentation after being challenged (control group). Interestingly, MOS supplementation (600 and 800 mg kg^-1^) performed the obvious reduction of DNA fragmentation. In **Figure 3B**, our results showed that compared with the control, the pro-apoptotic factors, *Caspase-3* (0.67-fold change), *Caspase-7* (0.31-fold change)*, FasL* (0.44-fold change), *BAX* (0.51-fold change), and *p38MAPK* (0.61-fold change), were significantly downregulated with optimal MOS supplementation up to 400 mg kg^-1^ (*P* < 0.05), and *Caspase-2* (0.62-fold change), *Caspase-8* (0.61-fold change), *Caspase-9* (0.68-fold change), and *Apaf-1* (0.75-fold change) were significantly downregulated with optimal MOS supplementation up to 600 mg kg^-1^ (*P* < 0.05); then all of them followed a gradual upward trend with the increase in MOS (600–1,000 mg kg^-1^ or 800–1,000 mg kg^-1^). The anti-apoptotic factors, *Bcl-2* (1.73-fold change), *IAP* (1.83-fold change), and *Mcl-1* (1.62-fold change), were significantly upregulated with optimal MOS supplementation up to 400 mg kg^-1^ (*P* < 0.05), and then all of them followed a gradual downward trend with the increase in MOS (600–1,000 mg kg^-1^) compared with the control group. However, we found that the MOS supplementation did not affect *JNK* mRNA levels. These data suggest that the MOS could effectively inhibit fish skin excessive apoptosis caused by *A. hydrophila*.

The tight junction proteins contribute to the skin barrier function (47). In **Figure 3C**, our results showed that compared with the control, most TJ protein genes, *ZO-1* (1.95-fold change), *ZO-2* (1.80-fold change), *Occludin* (1.87-fold change), *Claudin-3c* (1.56-fold change), *Claudin-7b* (1.73-fold change), *Claudin-11* (1.74-fold change), *Claudin-12* (1.61-fold change), *Claudin-15a* (1.77-fold change), and *Claudin-15b* (1.39-fold change), were significantly upregulated with optimal MOS supplementation up to 400 mg kg^-1^ (*P* < 0.05), and *Claudin-c* (1.83-fold change)*, Claudin-f* (1.93-fold change), and *Claudin-7a* (1.46-fold change) were significantly upregulated with optimal MOS supplementation up to 600 mg kg^-1^ (*P* < 0.05); then all of them followed a gradual downward trend with the increase in MOS (600–1,000 mg kg^-1^ or 800–1,000 mg kg^-1^). Furthermore, the key regulation molecules *MLCK* mRNA levels (0.58-fold change) were significantly downregulated with optimal MOS supplementation up to 400 mg kg^-1^ (*P* > 0.05) and plateaued with the increase in MOS (600–1,000 mg kg^-1^) compared with the control group. We also found that the MOS supplementation did not affect *Claudin-b* mRNA levels. These data suggest that MOS could enhance tight junctions of fish skin under *A. hydrophila* challenge.

### Skin Immune Barrier Function Gene Expression

To investigate the effect of MOS on fish skin immune barrier function with *A. hydrophila* challenge, the mRNA expression of the pro-inflammatory cytokines and anti-inflammatory cytokines and key signaling molecule gene was examined by real-time RT-PCR (**Figure 3D**). As is well known, inflammatory cytokines are crucial for fighting off infections and are involved in immune responses (48). In **Figure 3D**, compared with the control, the expression of pro-inflammatory cytokines, *IL-1β* (0.48-fold change), *TNF-α* (0.50-fold change), *IL-6* (0.57-fold change), *IL-12p40* (0.48-fold change), *IL-15* (0.43-fold change), and *IL-17D* (0.47-fold change), was significantly downregulated with MOS supplementation up to 400 mg kg^-1^ (*P* < 0.05), that of *IFNγ2* (0.65-fold change) and *IL-12p35* (0.47-fold change) was significantly downregulated with MOS supplementation up to 600 mg kg^-1^ (*P* < 0.05), and *IL-8* (0.58-fold change) was significantly downregulated with MOS supplementation up to 800 mg kg^-1^ (*P* < 0.05), followed by a gradual upward trend or plateau with the increase in MOS (600–1,000 mg kg^-1^). Besides, the anti-inflammatory cytokine factors *IL-4/13A* (1.64-fold change) and *IL-11* (1.66-fold change) were significantly upregulated with MOS supplementation up to 400 mg kg^-1^ (*P* > 0.05), and *TGFβ1* (1.76-fold change), *TGF-β2* (1.42-fold change), and *IL-10* (1.79-fold change) were significantly upregulated with MOS supplementation up to 600 mg kg^-1^ (*P* > 0.05), followed by a gradual downward trend with the increase in MOS (600–1,000 mg kg^-1^ or 800–1,000 mg kg^-1^), compared with the control group. Our results showed that the MOS supplementation did not affect *IL-4/13B* mRNA levels.

Many inflammatory cytokines could be mediated by NFκB and the TOR signaling pathway (29, 49). The present study displayed that compared with the control, the expression of inflammatory signal molecular-related genes, *NFκBp65* (0.57-fold change) and *4E-BP2* (0.59-fold change), was significantly downregulated with MOS supplementation up to 400 mg kg^-1^, that of *NFκBp52* (0.55-fold change), *c-Rel* (0.61-fold change), and *4E-BP1* (0.58-fold change) was significantly downregulated with MOS supplementation up to 600 mg kg^-1^, and that of *IKKβ* (0.75-fold change) and *IKKγ* (0.64-fold change) was significantly downregulated with MOS supplementation up to 800 mg kg^-1^, followed by a gradual upward trend with the increase in MOS (600–1,000 mg kg^-1^). Besides, *IκBα* (1.98-fold change) and *TOR* (2.11-fold change) were significantly upregulated with MOS supplementation up to 400 mg kg^-1^, and *S6K1* (1.92-fold change) was significantly upregulated with MOS supplementation up to 600 mg kg^-1^, followed by a gradual downward trend with the increase in MOS (600–1,000 mg kg^-1^), compared with the control group. Our results showed that the MOS supplementation did not affect *IKKα* mRNA levels. These results suggest that MOS is involved in the regulation of inflammatory cytokines under *A. hydrophila* challenge.

### Correlation Analysis

**Figure 4:**
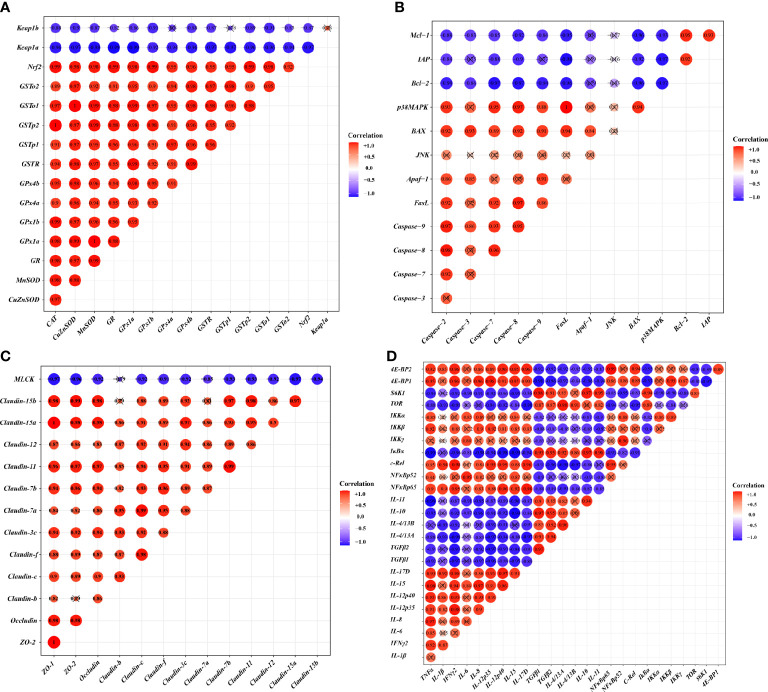
Correlation analysis of parameters in the skin of on-growing grass carp after infection of *Aeromonas hydrophila*. Antioxidant **(A)**, apoptosis **(B)**, TJs **(C)**, and immune **(D)** of on-growing grass carp after infection of *Aeromonas hydrophila*.

To investigate the correlation between the expression of genes related to the skin barrier function and the signal molecules involved in regulation, correlation analysis was performed. **Figures 4A-D** provides the diagram of the correlation analysis. These data showed the gene expression correlation analyses of physic barrier-related parameters and immune barrier-related parameters. Gene expression of studied antioxidant enzymes revealed a positive correlation with *Nrf2* mRNA levels, whereas *Keap1a* and *Keap1b* revealed a negative correlation. Gene expression of the studied pro-apoptotic factor showed a positive correlation with p38MAPK, whereas the anti-apoptotic factor showed a negative correlation. Gene expression of studied TJ proteins (except *Claudin-b*) showed a negative correlation with *MLCK*. Besides, gene expression of studied pro-inflammatory cytokine factors presented a positive correlation with *NFκB*, and the anti-inflammatory cytokine presented a positive correlation with *TOR*.

### Key Role Protein Levels of Skin Barrier Function

**Figure 5:**
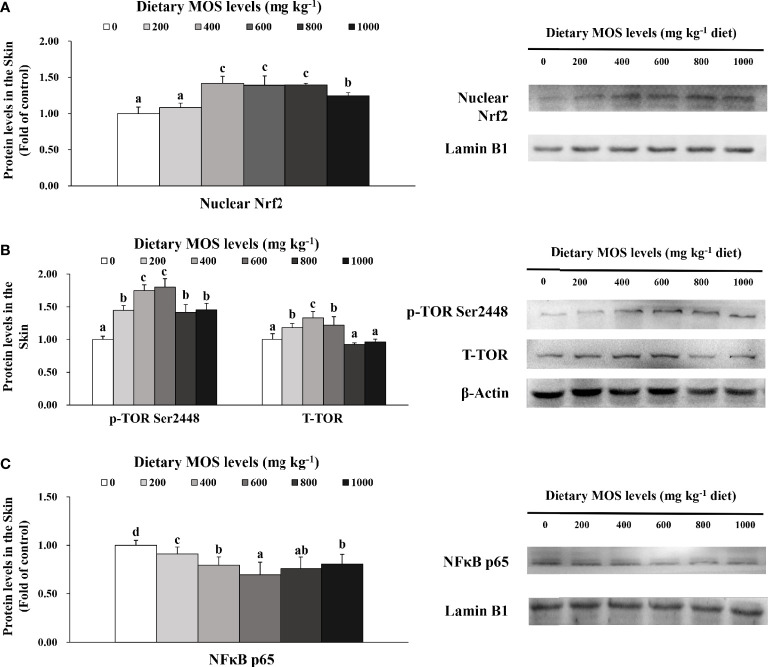
Western blot analysis of nuclear Nrf2 **(A)**, p-TOR Ser2448 **(B)** and NFκBp65 **(C)** protein levels in the skin of on-growing grass carp after infection of *Aeromonas hydrophila*. Data represent means of three fish in each group, error bars indicate S.D. Values having different letters are significantly different (*P* < 0.05).

To verify the results of skin barrier gene expression, we further performed Western blot analysis to test several key regulatory signaling molecules. The protein expression of Nrf2, TOR, and NFκB p65 in the skin of fish is exhibited in **Figures 5A–C**, respectively.

Compared with the control group, the nuclear Nrf2 (1.42-fold change) in the skin of fish was elevated with MOS supplementation up to 400 mg kg^-1^ (*P* > 0.05) and then plateaued with MOS supplementation up to 1,000 mg kg^-1^. Besides, fish fed with 600 and 400 mg kg^-1^ MOS presented the maximum p-TOR Ser2448 (1.80-fold change) and total TOR (T-TOR) (1.33-fold change) expression (*P* < 0.05), respectively, and then gradually decreased with MOS supplementation up to 1,000 mg kg^-1^ compared with the control group. With dietary MOS supplementation up to 600 mg kg^-1^, NFκB p65 expression (0.70-fold change) weakened obviously (*P* < 0.05) and then gradually increased with MOS supplementation up to 1,000 mg kg^-1^. As expected, these results of protein expression were consistent with those of gene expression.

## Discussion

This research used the same growth trial from our previous work in grass carp (35), which is a part of a larger study conducted to investigate the protective effect of fish skin barrier function by MOS supplementation. Our previous works have demonstrated that optimal MOS supplementation could promote fish growth and improve multiple functional organs (such as intestine, head-kidney, and spleen) health (35, 37). As is well known, fish growth and development are closely related to skin health (1). Therefore, to investigate the effects of prebiotics on fish skin health, we conducted relevant experiments based on previous studies.

### MOS Supplementation Enhanced Skin Disease Resistance

As is well known, skin health is mainly reflected by disease resistance (50). *Aeromonas hydrophila* is one of the most common pathogenic microorganisms associated with the aquatic environment, which could cause skin lesions in fish (51). In this study, our results displayed that optimal MOS (400 mg kg^-1^) could decrease skin lesion morbidity (8.27%) after being challenged while the control group caused skin lesion morbidity (14.40%), indicating that MOS supplementation enhanced fish resistance against skin lesions. Our data also showed that MOS supplementation attenuated skin hemorrhages and lesions, which suggested that MOS supplementation enhanced the ability to resist *A. hydrophila* invasion. Based on the quadratic regression analysis, the recommend suitable MOS supplementation against skin lesions morbidity was estimated to be 508.2 mg kg^-1^. Generally, skin health is closely related to physical barriers and immune barriers in fish (1). Therefore, at first, we investigated the effects of MOS supplementation on physical barrier function in the skin of on-growing grass carp.

### MOS Supplementation Enhanced Skin Physical Barrier Function

As mention above, the physical barrier function of the skin is related to cellular integrity and intercellular integrity, which were related to antioxidant capacity, apoptosis, and tight junction. Generally, MDA and PC were usually recognized to reflect the level of cell damage resulting from reactive oxygen (ROS) metabolites, which could be reduced by the antioxidant system (52, 53). We found that optimal MOS dosage decreased the biomarker content of oxidative damage of lipid and protein, whereas it enhanced the antioxidant enzyme activities. These data implied that MOS supplementation enhanced the antioxidant capacity to inhibit oxidative damage in fish skin. In general, antioxidant enzyme activities were strongly associated with their corresponding mRNA gene expression (54). We found that antioxidant enzymes and related isoform gene expression were upregulated by optimal MOS supplementation in the skin, indicating that MOS-enhanced activity of the antioxidant enzyme might be partly related to the upregulation of their mRNA levels. To our knowledge, Nrf2 is a major factor accounting for promoting the expression of various antioxidant enzyme genes to defend against oxidative stress, which is degenerated by Keap1 in the nucleus (55, 56). A study on mice liver showed that the Nrf2 protein level in the nucleus could evaluate the nuclear translocation of Nrf2 (57). Our result showed that MOS supplementation upregulated *Nrf2* and downregulated *Keap1a* (rather than *Keap1b*) and increased the protein levels of nucleus Nrf2, suggesting that MOS supplementation activated the Nrf2 signaling pathway by the activation of Nrf2 nuclear translocation in the skin. Notably, we found that MOS only downregulated the *Keap1a* expression in the skin, which might be partly relevant to threonine. A study on piglets revealed that threonine absorbed from the intestine could be enhanced by MOS supplementation (58). Our lab previously has confirmed that threonine has no influence on *Keap1b* gene expression in the grass carp gill (59). Thus, these data might partially support our hypothesis. However, the specific mechanism needs further investigation. In addition, a study reported that excessive oxidative damage could induce cell apoptosis in MN9D cells (60). Therefore, we further examined the effects of MOS supplementation on fish skin apoptosis.

Apoptosis, a tightly controlled physiological process, and internal environment homeostasis, plays important roles not only in the normal development and homeostasis of organisms but also in the pathogenesis of bacterial infections (61). However, excessive apoptosis could destroy the physical barrier of the skin in fish (62). In mammals, there are two major apoptosis pathways, the death receptor pathway (FasL/caspase-8) and the mitochondria pathway [(Bcl-2, Mcl-1, and Bax)/Apaf-1/caspase-9], which were modulated by signal molecule p38MAPK and JNK (63–65). These two apoptosis pathways converge on caspase-3 activation, which is the key apoptotic protein. As is well known, the apoptosis-related protein includes the apoptotic promoter (caspase-8 and caspase-9) and effector (caspase-3 and caspase-7). In addition, DNA fragmentation is a hallmark of apoptosis (66). The visualization of apparent index results clearly showed that the level of apoptosis was significantly reduced with MOS supplementation. Our gene expression results also displayed that the optimal MOS supplementation could suppress the excess apoptosis process under-challenged, which was partly associated with p38MAPK (not JNK), leading to the inhibition of both apoptosis pathways in fish skin. As mentioned above, intercellular structure integrity also played a crucial role in the physical barrier, which is associated with TJ proteins (67). Thus, we next examined the influences of MOS supplementation on TJs as well as the related signaling pathway in fish skin.

The intercellular junction complex function has maintained the integrity of the skin barrier, which mainly consists of TJ proteins (68, 69). It has been reported that inhibition of *MLCK* expression could improve epithelial TJ barrier function in Caco-2 cells (70). Our result displayed that optimal MOS upregulated the expression of most of the tight junction proteins (except *claudin-b*) and downregulated *MLCK*, suggesting that MOS improved tight junction partly by inhibiting the MLCK signaling pathway. We surprisingly found that MOS did not affect *claudin-b* gene expression, which could involve both IL-6 and cortisol. Our result exhibited that MOS supplementation could downregulate *IL-6* gene expression. Steensberg et al. (71) confirmed that IL-6 could increase the content of cortisol in humans. Studies showed that cortisol did not affect claudin-b mRNA levels in the gill epithelial cell of pufferfish and goldfish (72, 73), which supports our hypothesis. However, determining the underlying mechanism warrants further investigation.

### MOS Supplementation Enhanced Skin Immune Barrier Function

To our knowledge, the existence and function of the secretory cell in teleost skin (such as mucous goblet cells, squamous cells, pigment cells, and so on) have been confirmed and provided the first line of defense against pathogen invasion (1, 74). The mucus secreted by these cells contains a large number of antimicrobial substances (75, 76). Previous studies have demonstrated that MOS can increase the LZ activity and bactericidal activity in the skin of greater amberjack (13). The present study focuses on antibacterial compounds, and the results revealed that MOS could promote LZ production in the skin of grass carp, agreeing with previous findings in the skin of greater amberjack. Coincidentally, we also found a study that showed that other prebiotics also has antimicrobial properties, which reported the antimicrobial ability to be enhanced in the skin of Caspian white fish (*Rutilus frisii kutum*) with xylooligosaccharide (77). These interesting results partly reflect the commonality of prebiotic to improve skin antimicrobial capacity. In addition, the skin immune function is closely related to the inflammatory response mediated by cytokines (14). Thus, we next examined the effects of MOS supplementation on fish skin immune barrier function.

In the immune system, there is a dynamic balance between pro-inflammatory cytokines and ant-inflammatory cytokines. The imbalance of inflammatory cytokines caused by external stimuli (pathogenic bacteria) is one of the causes of the excessive inflammatory response (78). A study on channel catfish, *Ictalurus punctatus*, revealed that Actigen^®^ (a commercial MOS product from Alltech) could improve inflammatory cytokine balance in multiple mucosal immune organs by using RNA-seq, indicating that MOS additives may provide protection extending beyond the intestine to surface mucosa (79). As we expected, our result displayed that optimal MOS dosage downregulated pro-inflammatory cytokine expression; in contrast to the former, the anti-inflammatory cytokine (except *IL-4/13B*) expression was upregulated, indicating that MOS supplementation attenuated the inflammation in fish skin. Notably, part of these data differed with other similar studies (parasite challenged) in the skin of greater amberjack, which found that *TNFα*, *IL-1β*, *IFNγ*, and *IL-8* were upregulated by MOS supplementation (2 g kg^-1^) (13). Differences in species, MOS purity, and challenged type might account for this disparity. Notably, another interesting result showed that dietary MOS only upregulated *IL-4/13A* expression in the skin. This phenomenon might be associated with the content of phosphorus. A study on weaned piglets confirmed that MOS increased the digestibility of phosphorus (30). Past work in our lab has confirmed that phosphorus has no effect on the *IL-4/13B* expression, and our results also showed that dietary MOS did the same effect on *IL-4/13B* expression (32). Thus, we speculated that MOS supplementation upregulates the *IL-4/13A* (rather than IL-4/13B), which might relate to improving the digestibility of phosphorus, thus leading to a disposition of only upregulated *IL-4/13A* in fish.

As we all know, the pro-inflammatory cytokines could be activated by the NFκB family of transcription factors (such as NFκB p65, p52, and c-Rel), which required a sequestering protein named IκBα that could be catalyzed by the IKK complex (IKKα, IKKβ, and IKKγ) (80, 81). We found that optimal MOS supplementation downregulated *NFκB*-related signal molecule (rather than *IKKα*) gene expression and decreased the protein levels of NFκB p65, suggesting that MOS supplementation activated the NFκB signaling pathway by decreasing the nuclear NFκB p65 protein expression in the skin. Interestingly, what is noteworthy of this study is that MOS supplementation did not have influence on IKKα in the skin; the possible reasons for this difference might be due to TNF-α and PKCζ. Our result revealed that MOS could downregulate TNF-α expression. A study on rat showed that downregulated TNF-α expression could decrease the activity of PKCζ (82), which could downregulate IKKβ and IKKγ (rather than IKKα) expression in Kupffer cells, and did not have an effect on IKKα expression (83), supporting our hypothesis. However, the underlying molecular mechanism is still unknown and warrants further investigation. In addition, it has been reported that anti-inflammatory cytokines could be modulated by the mTOR/(S6K1, 4EBP-1) signaling cascades in humans (84). One study on rainbow trout reported that the phosphorylation of TOR on residue Ser2448 can be used to monitor the activation of TOR signaling (85). We found that MOS supplementation downregulated *4EBP-1* and *4EBP-2* gene expression and upregulated *TOR* and *S6K1* expression, and increased the protein levels of TOR and p-TOR Ser2448, suggesting that MOS supplementation upregulated the anti-inflammatory cytokine mRNA levels partly due to the activation of the TOR signaling pathway cascades in fish skin.

In summary, the current work presented a clear outline of dietary MOS enhanced fish skin immune barrier and physical barrier function after infection with *A. hydrophila*. Our study confirmed that dietary MOS supplementation could improve the status of skin health, as demonstrated by the following findings (1): MOS supplementation enhanced the immune barrier function *via* increasing the skin disease resistance, producing antibacterial compounds and immunoglobulins, upregulating anti-inflammatory cytokines (except *IL-4/13B*), and downregulating pro-inflammatory cytokines gene expression (2). MOS supplementation protected the physical barrier function *via* increasing the antioxidant capacity, inhibited excessive apoptosis, and enhanced the tight junction barriers (except *claudin-b*). Moreover, MOS supplementation improved fish physical and immune barrier function by modulating multiple signaling pathways (such as Nrf2, TOR, NFκB, and so on).

## Data Availability Statement

The original contributions presented in the study are included in the article/**Supplementary Material**. Further inquiries can be directed to the corresponding author.

## Ethics Statement

The animal study was reviewed and approved by Laboratory Animals Care and Use of Animal Nutrition Institute of Sichuan Agricultural University.

## Author Contributions

ZyL performed formal analysis, investigation and writing original draft. LF performed conceptualization, funding acquisition and supervision. W-DJ performed data curation, validation, project administration and writing review & editing. PW performed conceptualization, methodology, validation, data curation and project administration. YL and JJ performed project administration. S-YK, LT, S-WL, C-BZ performed resources. X-QZ performed conceptualization, designed experiment, supervision and funding acquisition. All authors contributed to the article and approved the submitted version.

## Funding

This research was financially supported by the National Key R&D Program of China (2019YFD0900200 and 2018YFD0900400), the National Natural Science Foundation of China for Outstanding Youth Science Foundation (31922086), and the Young Top-Notch Talent Support Program, Supported by China Agriculture Research System of MOF and MARA (CARS-45), and supported by Sichuan Science and Technology Program (2019YFN0036). The authors would like to thank the personnel of these teams for their kind assistance.

## Conflict of Interest

S-YK, LT, S-WL, X-AL and C-BZ were employed by Sichuan Animtech Feed Co. Ltd.

The remaining authors declare that the research was conducted in the absence of any commercial or financial relationships that could be construed as a potential conflict of interest.

## Publisher’s Note

All claims expressed in this article are solely those of the authors and do not necessarily represent those of their affiliated organizations, or those of the publisher, the editors and the reviewers. Any product that may be evaluated in this article, or claim that may be made by its manufacturer, is not guaranteed or endorsed by the publisher.

## Supplementary Material

Supplementary Figure 1 Antioxidative activities of MOS with different levels. **(A)** 2,2-diphenyl-1-picrylhydrazyl (DPPH) radical scavenging activity. **(B) ·**O_2_ radical scavenging activity. **(C) ·**OH scavenging activity.

Supplementary Figure 2 DNA fragmentation analysis in the skin of on-growing grass carp after infection of *Aeromonas hydrophila*. Lane 1: maker. Lane 2- Lane 6: levels of dietary MOS were 0, 200, 400, 600, 800 and 1000 mg kg^-1^, respectively. This experiment was repeated three times with similar results achieved.
