# Supplementary figures and images for: Mannan Oligosaccharides Application: Multipath Restriction From Aeromonas hydrophila Infection in the Skin Barrier of Grass Carp (Ctenopharyngodon idella)
Source: Front Immunol. 2021 Oct 18;12:742107. doi: 10.3389/fimmu.2021.742107 (PMC8559429; doi:10.3389/fimmu.2021.742107)

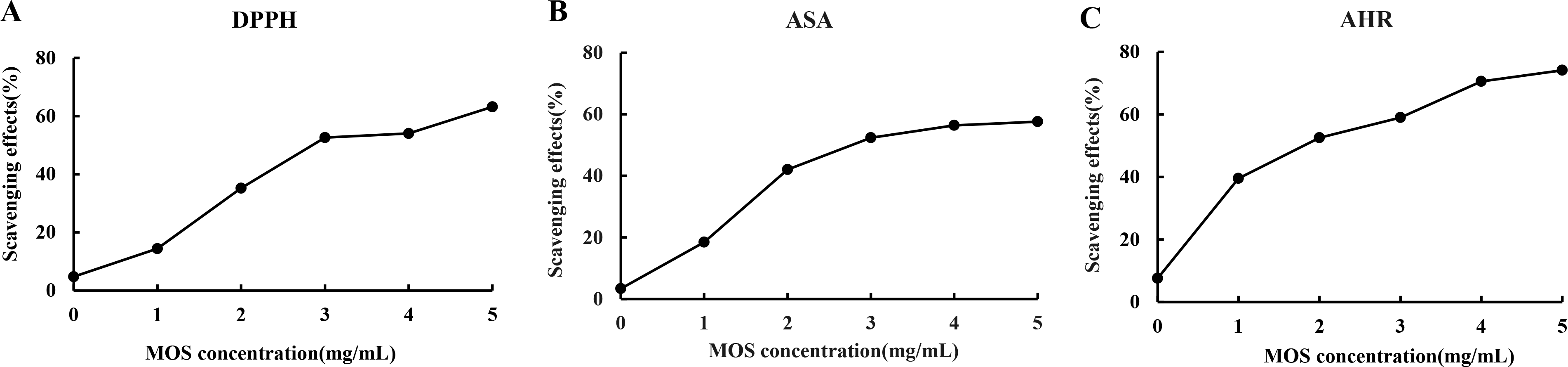

Supplement: Supplementary Figure 1 — Antioxidative activities of MOS with different levels. (A) 2,2-diphenyl-1-picrylhydrazyl (DPPH) radical scavenging activity. (B) ·O2 radical scavenging activity. (C) ·OH scavenging activity. [file Image_1.tif]

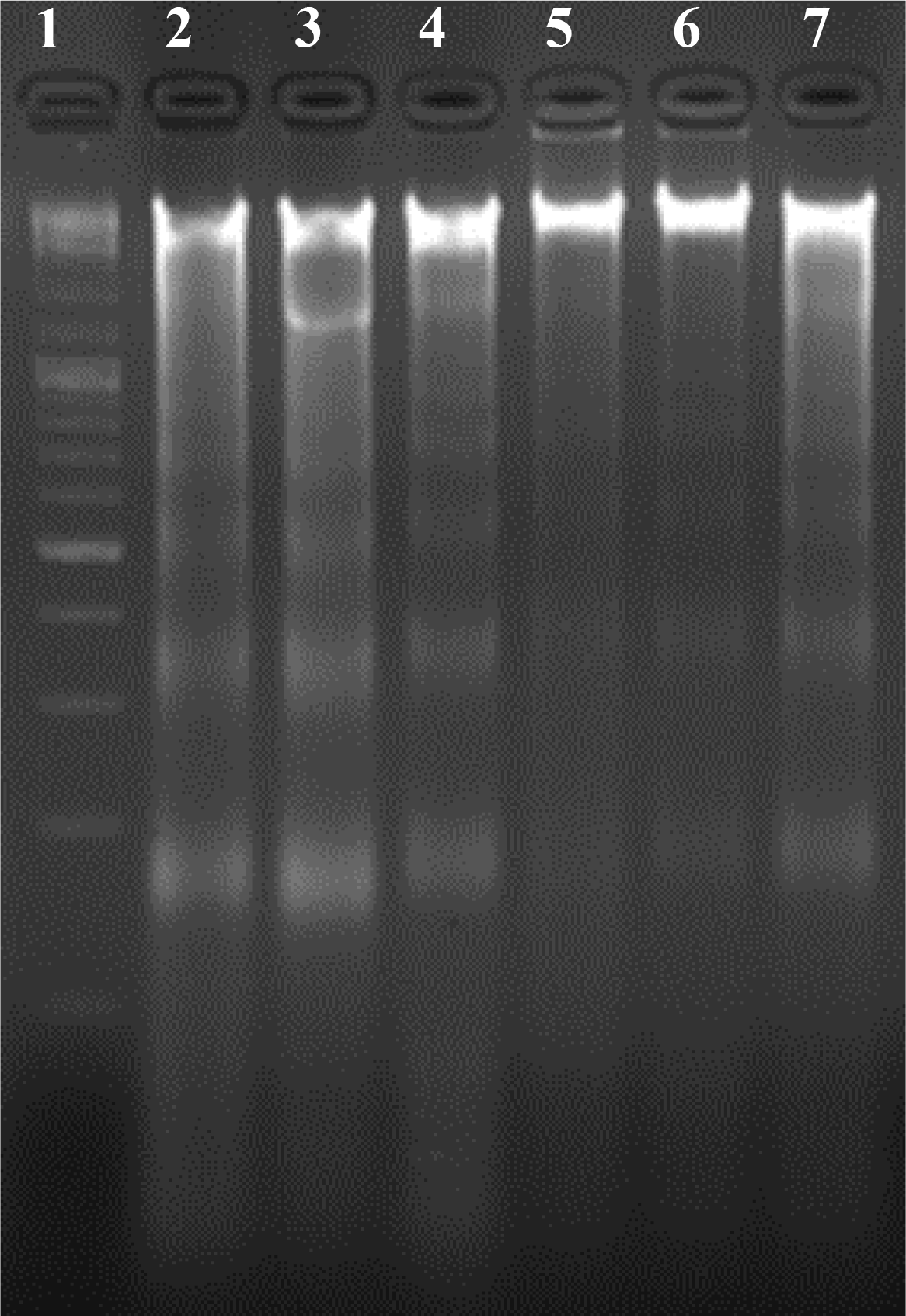

Supplement: Supplementary Figure 2 — DNA fragmentation analysis in the skin of on-growing grass carp after infection of Aeromonas hydrophila. Lane 1: maker. Lane 2- Lane 6: levels of dietary MOS were 0, 200, 400, 600, 800 and 1000 mg kg-1, respectively. This experiment was repeated three times with similar results achieved. [file Image_2.tif]
