# Supplementary material for: Mannan Oligosaccharides Application: Multipath Restriction From Aeromonas hydrophila Infection in the Skin Barrier of Grass Carp (Ctenopharyngodon idella)
Source: Front Immunol. 2021 Oct 18;12:742107. doi: 10.3389/fimmu.2021.742107 (PMC8559429; doi:10.3389/fimmu.2021.742107)
Supplement: Supplementary file 3 [file Table_1.docx]

**Supplementary Table 1.** Composition and nutrient content of the diet.

| Ingredients | % | Nutrient content | % |
| --- | --- | --- | --- |
| Fish meal | 7.80 | Crude protein ^4^ | 28.69 |
| Gelatin | 6.00 | Crude lipid ^4^ | 5.36 |
| Soybean protein concentrated | 26.00 | n-3 ^4^ | 1.04 |
| Corn starch | 19.90 | n-6 ^4^ | 0.96 |
| α-starch | 24.00 | Available phosphorus ^4^ | 0.40 |
| Fish oil | 2.34 |  |  |
| Soybean oil | 1.81 |  |  |
| Cellulose | 5.00 |  |  |
| Ca (H_2_PO4)_2_ | 1.30 |  |  |
| Vitamin premix ^1^ | 1.00 |  |  |
| Mineral premix ^2^ | 2.00 |  |  |
| MOS premix ^3^ | 1.00 |  |  |
| Choline chloride (50%) | 1.00 |  |  |
| Ethoxyquin (30%) | 0.05 |  |  |
| DL-Met (99%) | 0.61 |  |  |
| L-Trp (99%) | 0.08 |  |  |
| Thr (98.5%) | 0.11 |  |  |

^1^ Per kilogram of vitamin premix (g kg^-1^): retinyl acetate (500,000 IU g^-1^), 0.39; cholecalciferol (500,000 IU g^-1^), 0.40; D, L-α-tocopherol acetate (50%), 23.23; menadione (22.9%), 0.83; cyanocobalamin (1%), 0.94; D-biotin (2%), 0.75; folic acid (95%), 0.42; thiamine nitrate (98%), 0.09; ascorhyl acetate (95%), 9.77; niacin (99%), 4.04; meso-inositol (98%), 19.39; Calcium-D-pantothenate (98%), 3.85; riboflavin (80%), 0.73; pyridoxine hydrochloride (98%), 0.62. All ingredients were diluted with corn starch to 1 kg.

^2^ Per kilogram of mineral premix (g kg^-1^): MnSO_4_⋅H_2_O (31.8% Mn), 2.6590; MgSO_4_⋅H_2_O (15.0% Mg), 200.0000; FeSO_4_⋅H_2_O (30.0% Fe), 12.2500; ZnSO_4_.H_2_O (34.5% Zn), 8.2460; CuSO_4_⋅5H_2_O (25.0% Cu), 0.9560; KI (76.9% I), 0.0650; Na_2_SeO_3_ (44.7% Se), 0.0168. All ingredients were diluted with corn starch to 1 kg.

^3^ MOS premix (mg kg^-1^): premix was added to obtain graded levels of MOS.

^4^ Crude protein and crude lipid content were measured value. Available phosphorus, n-3 and n-6 contents was referenced to [Lu et al. (2020a)](#_ENREF_41) and calculated according to NRC (2011).
