# Supplementary material for: Mannan Oligosaccharides Application: Multipath Restriction From Aeromonas hydrophila Infection in the Skin Barrier of Grass Carp (Ctenopharyngodon idella)
Source: Front Immunol. 2021 Oct 18;12:742107. doi: 10.3389/fimmu.2021.742107 (PMC8559429; doi:10.3389/fimmu.2021.742107)
Supplement: Supplementary file 4 [file Table_2.docx]

**Supplementary Table 2.** The analysis method of biomarker and enzymes activity related parameters

| Indices | Analysis method |
| --- | --- |
| Reactive oxygen species (ROS) | Chemical fluorescence method (Biyuntian, Shanghai, China). |
| Malondialdehyde (MDA) | The thiobarbituric acid (TBA) assay kit (Nanjing Jiancheng Institute, China) |
| 2,2-diphenyl-1-picrylhydrazyl (DPPH) | The DPPH radical scavenging activity reagent (Solarbio, Beijing, China) |
| Protein carbonyl (PC) | The 2, 4-dinitrophenylhydrazine (DNPH) reagent (Nanjing Jiancheng Institute, China) |
| Anti-superoxide anion (ASA) | The superoxide anion free radical detection Kit (Nanjing Jiancheng Institute, China) |
| Anti-hydroxy radical (AHR) | The hydroxyl free radical detection Kit (Nanjing Jiancheng Institute, China) |
| Superoxide dismutase (SOD) | The Hydroxylamine method (Nanjing Jiancheng Institute, China) |
| Catalase (CAT) | Visible light, commercial kit (Nanjing Jiancheng Institute, China) |
| Glutathione peroxidase (GPx) | Colorimetric method, commercial kit (Nanjing Jiancheng Institute, China) |
| Glutathione-S-transferase (GST) | Colorimetric method, commercial kit (Nanjing Jiancheng Institute, China) |
| Glutathione reductase (GR) | A commercial kit (Nanjing Jiancheng Institute, China) |
| Glutathione (GSH) | Spectrophotometric method, commercial kit (Nanjing Jiancheng Institute, China) |
| Protein concentrations | Coomassie brilliant blue method, commercial kit (Nanjing Jiancheng Institute, China) |
| Lysozyme (LZ) | Spectrophotometry, commercial kit (Nanjing Jiancheng Institute, China) |
| Acid phosphatase (ACP) | Spectrophotometry, commercial kit (Nanjing Jiancheng Institute, China) |
| Complement (C3) | The immunoturbidimetry kit (Yilikang Biotechnology co., Ltd., Zhejiang, China) |
| Complement (C4) | The immunoturbidimetry kit (Yilikang Biotechnology co., Ltd., Zhejiang, China) |
| Immunoglobulin (IgM) | The immunoturbidimetry kit (Yilikang Biotechnology co., Ltd., Zhejiang, China) |
