# Supplementary material for: Mannan Oligosaccharides Application: Multipath Restriction From Aeromonas hydrophila Infection in the Skin Barrier of Grass Carp (Ctenopharyngodon idella)
Source: Front Immunol. 2021 Oct 18;12:742107. doi: 10.3389/fimmu.2021.742107 (PMC8559429; doi:10.3389/fimmu.2021.742107)
Supplement: Supplementary file 5 [file Table_3.docx]

**Supplementary Table 3**. Real-time PCR primer sequences.

| **Target gene** | **Primer sequence (5’→3’)** | **Temperature(°C)** | **Accession number** |
| --- | --- | --- | --- |
| ***CuZnSOD*** | **Forward:** CGCACTTCAACCCTTACA | 61.5 | GU901214 |
|  | **Reverse:** ACTTTCCTCATTGCCTCC |  |  |
| ***MnSOD*** | **Forward:** ACGACCCAAGTCTCCCTA | 60.4 | GU218534 |
|  | **Reverse:** ACCCTGTGGTTCTCCTCC |  |  |
| ***CAT*** | **Forward:** GAAGTTCTACACCGATGAGG | 58.7 | FJ560431 |
|  | **Reverse:** CCAGAAATCCCAAACCAT |  |  |
| ***GPx1a*** | **Forward:** GGGCTGGTTATTCTGGGC | 61.5 | EU828796 |
|  | **Reverse:** AGGCGATGTCATTCCTGTTC |  |  |
| ***GPx1b*** | **Forward:** TTTTGTCCTTGAAGTATGTCCGTC | 60.3 | KT757315 |
|  | **Reverse:** GGGTCGTTCATAAAGGGCATT |  |  |
| ***GPx4a*** | **Forward:** TACGCTGAGAGAGGTTTACACAT | 60.4 | KU255598 |
|  | **Reverse:** CTTTTCCATTGGGTTGTTCC |  |  |
| ***GPx4b*** | **Forward:** CTGGAGAAATACAGGGGTTACG | 60.3 | KU255599 |
|  | **Reverse:** CTCCTGCTTTCCGAACTGGT |  |  |
| ***GSTR*** | **Forward:** TCTCAAGGAACCCGTCTG | 58.4 | EU107283 |
|  | **Reverse:** CCAAGTATCCGTCCCACA |  |  |
| ***GSTp1*** | **Forward:** ACAGTTGCCCAAGTTCCAG | 59.3 | KM112099 |
|  | **Reverse:** CCTCACAGTCGTTTTTTCCA |  |  |
| ***GSTp2*** | **Forward:** TGCCTTGAAGATTATGCTGG | 59.3 | KP125490 |
|  | **Reverse:** GCTGGCTTTTATTTCACCCT |  |  |
| ***GSTo1*** | **Forward:** GGTGCTCAATGCCAAGGGAA | 58.4 | KT757314 |
|  | **Reverse:** CTCAAACGGGTCGGATGGAA |  |  |
| ***GSTo2*** | **Forward:** CTGCTCCCATCAGACCCATTT | 61.4 | KU245630 |
|  | **Reverse:** TCTCCCCTTTTCTTGCCCATA |  |  |
| ***GR*** | **Forward:** GTGTCCAACTTCTCCTGTG | 59.4 | JX854448 |
|  | **Reverse:** ACTCTGGGGTCCAAAACG |  |  |
| ***Nrf2*** | **Forward:** CTGGACGAGGAGACTGGA | 62.5 | KF733814 |
|  | **Reverse:** ATCTGTGGTAGGTGGAAC |  |  |
| ***Keap1a*** | **Forward:** TTCCACGCCCTCCTCAA | 63.0 | KF811013 |
|  | **Reverse:** TGTACCCTCCCGCTATG |  |  |
| ***Keap1b*** | **Forward:** TCTGCTGTATGCGGTGGGC | 57.9 | KJ729125 |
|  | **Reverse:** CTCCTCCATTCATCTTTCTCG |  |  |
| ***FasL*** | **Forward:** AGGAAATGCCCGCACAAATG | 61.4 | KT445873 |
|  | **Reverse:** AACCGCTTTCATTGACCTGGAG |  |  |
| ***p38 MAPK*** | **Forward:** TGGGAGCAGACCTCAACAAT | 60.4 | KM112098 |
|  | **Reverse:** TACCATCGGGTGGCAACATA |  |  |
| ***JNK*** | **Forward:** ACAGCGTAGATGTGGGTGATT | 62.3 | KT757312 |
|  | **Reverse:** GCTCAAGGTTGTGGTCATACG |  |  |
| ***Bcl-2*** | **Forward:** AGGAAAATGGAGGTTGGGAT | 60.3 | JQ713862 |
|  | **Reverse:** CTGAGCAAAAAAGGCGATG |  |  |
| ***Mcl-1*** | **Forward:** TGGAAAGTCTCGTGGTAAAGCA | 58.4 | KT757307 |
|  | **Reverse:** ATCGCTGAAGATTTCTGTTGCC |  |  |
| ***Bax*** | **Forward:** CATCTATGAGCGGGTTCGTC | 60.3 | JQ793788 |
|  | **Reverse:** TTTATGGCTGGGGTCACACA |  |  |
| ***Apaf-1*** | **Forward:** AAGTTCTGGAGCCTGGACAC | 61.4 | KM279717 |
|  | **Reverse:** AACTCAAGACCCCACAGCAC |  |  |
| ***IAP*** | **Forward:** CACAATCCTGGTATGCGTCG | 58.4 | FJ593503.1 |
|  | **Reverse:** GGGTAATGCCTCTGGTGCTC |  |  |
| ***Caspase-2*** | **Forward:** CGCTGTTGTGTGTTTACTGTCTCA | 60.3 | KT757313 |
|  | **Reverse:** ACGCCATTATCCATCTCCTCTC |  |  |
| ***Caspase-3*** | **Forward:** GCTGTGCTTCATTTGTTTG | 55.9 | JQ793789 |
|  | **Reverse:** TCTGAGATGTTATGGCTGTC |  |  |
| ***Caspase-7*** | **Forward:** GCCATTACAGGATTGTTTCACC | 57.1 | KT625601 |
|  | **Reverse:** CCTTATCTGTGCCATTGCGT |  |  |
| ***Caspase-8*** | **Forward:** ATCTGGTTGAAATCCGTGAA | 59.0 | KM016991 |
|  | **Reverse:** TCCATCTGATGCCCATACAC |  |  |
| ***Caspase-9*** | **Forward:** CTGTGGCGGAGGTGAGAA | 59.0 | JQ793787 |
|  | **Reverse:** GTGCTGGAGGACATGGGAAT |  |  |
| ***Occludin*** | **Forward:** TATCTGTATCACTACTGCGTCG | 59.4 | KF193855 |
|  | **Reverse:** CATTCACCCAATCCTCCA |  |  |
| ***ZO-1*** | **Forward:** CGGTGTCTTCGTAGTCGG | 59.4 | KJ000055 |
|  | **Reverse:** CAGTTGGTTTGGGTTTCAG |  |  |
| ***ZO-2*** | **Forward:** TACAGCGGGACTCTAAAATGG | 60.3 | KM112095 |
|  | **Reverse:** TCACACGGTCGTTCTCAAAG |  |  |
| ***Claudin-b*** | **Forward:** GAGGGAATCTGGATGAGC | 57.0 | KF193860 |
|  | **Reverse:** ATGGCAATGATGGTGAGA |  |  |
| ***Claudin-c*** | **Forward:** GAGGGAATCTGGATGAGC | 59.4 | KF193859 |
|  | **Reverse:** CTGTTATGAAAGCGGCAC |  |  |
| ***Claudin-f*** | **Forward:** GCTGGAGTTGCCTGTCTTATTC | 57.1 | KM112097 |
|  | **Reverse:** ACCAATCTCCCTCTTTTGTGTC |  |  |
| ***Claudin-3c*** | **Forward:** ATCACTCGGGACTTCTA | 57.0 | KF193858 |
|  | **Reverse:** CAGCAAACCCAATGTAG |  |  |
| ***Claudin-7a*** | **Forward:** ACTTACCAGGGACTGTGGATGT | 59.3 | KT625604 |
|  | **Reverse:** CACTATCATCAAAGCACGGGT |  |  |
| ***Claudin-7b*** | **Forward:** CTAACTGTGGTGGTGATGAC | 59.3 | KT445866 |
|  | **Reverse:** AACAATGCTACAAAGGGCTG |  |  |
| ***Claudin-11*** | **Forward:** TCTCAACTGCTCTGTATCACTGC | 62.3 | KT445867 |
|  | **Reverse:** TTTCTGGTTCACTTCCGAGG |  |  |
| ***Claudin-12*** | **Forward:** CCCTGAAGTGCCCACAA | 55.4 | KF998571 |
|  | **Reverse:** GCGTATGTCACGGGAGAA |  |  |
| ***Claudin-15a*** | **Forward:** TGCTTTATTTCTTGGCTTTC | 59.0 | KF193857 |
|  | **Reverse:** CTCGTACAGGGTTGAGGTG |  |  |
| ***Claudin-15b*** | **Forward:** AGTGTTCTAAGATAGGAGGGGAG | 62.3 | KT757304 |
|  | **Reverse:** AGCCCTTCTCCGATTTCAT |  |  |
| ***MLCK*** | **Forward:** GAAGGTCAGGGCATCTCA | 53.0 | KM279719 |
|  | **Reverse:** GGGTCGGGCTTATCTACT |  |  |
| ***TNF-α*** | **Forward:** CGCTGCTGTCTGCTTCAC | 58.4 | HQ696609 |
|  | **Reverse:** CCTGGTCCTGGTTCACTC |  |  |
| ***IFN-γ2*** | **Forward:** TGTTTGATGACTTTGGGATG | 60.4 | JX657682 |
|  | **Reverse:** TCAGGACCCGCAGGAAGAC |  |  |
| ***IL-1β*** | **Forward:** AGAGTTTGGTGAAGAAGAGG | 57.1 | JQ692172 |
|  | **Reverse:** TTATTGTGGTTACGCTGGA |  |  |
| ***IL-6*** | **Forward:** CAGCAGAATGGGGGAGTTATC | 62.3 | KC535507.1 |
|  | **Reverse:** CTCGCAGAGTCTTGACATCCTT |  |  |
| ***IL-8*** | **Forward:** ATGAGTCTTAGAGGTCTGGGT | 60.3 | JN663841 |
|  | **Reverse:** ACAGTGAGGGCTAGGAGGG |  |  |
| ***IL-10*** | **Forward:** AATCCCTTTGATTTTGCC | 61.4 | HQ388294 |
|  | **Reverse:** GTGCCTTATCCTACAGTATGTG |  |  |
| ***IL-11*** | **Forward:** GGTTCAAGTCTCTTCCAGCGAT | 57.0 | KT445870 |
|  | **Reverse:** TGCGTGTTATTTTGTTCAGCCA |  |  |
| ***IL-12p35*** | **Forward:** TGGAAAAGGAGGGGAAGATG | 55.4 | KF944667.1 |
|  | **Reverse:** AGACGGACGCTGTGTGAGTGTA |  |  |
| ***IL-12p40*** | **Forward:** ACAAAGATGAAAAACTGGAGGC | 59.0 | KF944668.1 |
|  | **Reverse:** GTGTGTGGTTTAGGTAGGAGCC |  |  |
| ***IL-15*** | **Forward:** CCTTCCAACAATCTCGCTTC | 61.4 | KT445872 |
|  | **Reverse:** AACACATCTTCCAGTTCTCCTT |  |  |
| ***IL-17D*** | **Forward:** GTGTCCAGGAGAGCACCAAG | 62.3 | KF245426.1 |
|  | **Reverse:** GCGAGAGGCTGAGGAAGTTT |  |  |
| ***IL-4/13A*** | **Forward:** CTACTGCTCGCTTTCGCTGT | 55.9 | KT445871 |
|  | **Reverse:** CCCAGTTTTCAGTTCTCTCAGG |  |  |
| ***IL-4/13B*** | **Forward:** TGTGAACCAGACCCTACATAACC | 55.9 | KT625600 |
|  | **Reverse:** TTCAGGACCTTTGCTGCTTG |  |  |
| ***TGF-β1*** | **Forward:** TTGGGACTTGTGCTCTAT | 55.9 | EU099588 |
|  | **Reverse:** AGTTCTGCTGGGATGTTT |  |  |
| ***TGF-β2*** | **Forward:** TACATTGACAGCAAGGTGGTG | 55.9 | KM279716 |
|  | **Reverse:** TCTTGTTGGGGATGATGTAGTT |  |  |
| ***NF-κB p65*** | **Forward:** GAAGAAGGATGTGGGAGATG | 62.3 | KJ526214 |
|  | **Reverse:** TGTTGTCGTAGATGGGCTGAG |  |  |
| ***NF-κB p52*** | **Forward:** TCAGTGTAACGACAACGGGAT | 58.4 | KM279720 |
|  | **Reverse:** ATACTTCAGCCACACCTCTCTTAG |  |  |
| ***c-Rel*** | **Forward:** GCGTCTATGCTTCCAGATTTACC | 59.3 | KT445865 |
|  | **Reverse:** ACTGCCACTGTTCTTGTTCACC |  |  |
| ***IκBα*** | **Forward:** TCTTGCCATTATTCACGAGG | 62.3 | KJ125069 |
|  | **Reverse:** TGTTACCACAGTCATCCACCA |  |  |
| ***IKKα*** | **Forward:** GGCTACGCCAAAGACCTG | 60.3 | KM279718 |
|  | **Reverse:** CGGACCTCGCCATTCATA |  |  |
| ***IKKβ*** | **Forward:** GTGGCGGTGGATTATTGG | 60.3 | KP125491 |
|  | **Reverse:** GCACGGGTTGCCAGTTTG |  |  |
| ***IKKγ*** | **Forward:** AGAGGCTCGTCATAGTGG | 58.4 | KM079079 |
|  | **Reverse:** CTGTGATTGGCTTGCTTT |  |  |
| ***TOR*** | **Forward:** TCCCACTTTCCACCAACT | 61.4 | JX854449 |
|  | **Reverse:** ACACCTCCACCTTCTCCA |  |  |
| ***S6K1*** | **Forward:** TGGAGGAGGTAATGGACG | 54.0 | EF373673 |
|  | **Reverse:** ACATAAAGCAGCCTGACG |  |  |
| ***4E-BP1*** | **Forward:** GCTGGCTGAGTTTGTGGTTG | 60.3 | KT757305 |
|  | **Reverse:** CGAGTCGTGCTAAAAAGGGTC |  |  |
| ***4E-BP2*** | **Forward:** CACTTTATTCTCCACCACCCC | 60.3 | KT757306 |
|  | **Reverse:** TTCATTGAGGATGTTCTTGCC |  |  |
| ***GAPDH*** | **Forward:** GTTACAAGGGAGAAGTTCACCAT | 58.0 | GQ266395 |
|  | **Reverse:** CCGGTAGACTCGACTACATACAG |  |  |
| ***β-actin*** | **Forward:** GGCTGTGCTGTCCCTGTA | 61.4 | M25013 |
|  | **Reverse:** GGGCATAACCCTCGTAGAT |  |  |

^1^ *CuZnSOD*, copper, zinc superoxide dismutase; *MnSOD*, manganese superoxide dismutase; *CAT*, catalase; *GPx*, glutathione peroxidase; *GST*, glutathione-*S*-transferase; *GR*, glutathione reductase; *Nrf2*, NF-E2-related factor 2; *Keap1*, Kelch-like-ECH-associated protein 1; *GAPDH*, glyceraldehyde-3-phosphate dehydrogenase. *ZO*, zonula occludens; *MLCK*, myosin light chain kinase; *FasL*, fatty acid synthetase ligand; *p38 MAPK*, p38 mitogen-activated protein kinase; *JNK*, c-Jun Nterminal protein kinase; *Bcl-2*, B-cell lymphoma protein-2; *Mcl-1*, myeloid cell leukemia-1; *Bax*, Bcl-2 associated X protein; *Apaf-1*, apoptotic protease activating factor-1; *IAP*, inhibitor ofapoptosis proteins; *Caspase*, cysteinyl aspartic acid-protease; *TNF-α*, tumor necrosis factor α; *IFN-γ2*, interferon γ2; *IL*, interleukin; *TGF-β*, transforming growth factor β; *NF-κB p65*, nuclear factor kappa B p65; *NF-κB p52*, nuclear factor kappa B p52; *IκBα*, inhibitor of κBα; *IKK*, IκB kinase; *TOR*, target of rapamycin; *S6K1*, ribosomal protein S6 kinase 1; *4E-BP*, eIF4E-binding protein.
