# Supplementary material for: Mannan Oligosaccharides Application: Multipath Restriction From Aeromonas hydrophila Infection in the Skin Barrier of Grass Carp (Ctenopharyngodon idella)
Source: Front Immunol. 2021 Oct 18;12:742107. doi: 10.3389/fimmu.2021.742107 (PMC8559429; doi:10.3389/fimmu.2021.742107)
Supplement: Supplementary file 6 [file Table_4.docx]

**Supplementary Table 4.** The information of antibodies (Western blot)

| Indices | Host | Source | Catalog No. | Dilution for WB |
| --- | --- | --- | --- | --- |
| Nucleus Nrf2 | Rabbit | Abcam (Cambridge, MA, USA)) | ab31163 | 1:1000 |
| β-actin | Rabbit | Affinity (Golden, Colorado, USA) | AF7018 | 1:3000 |
| LaminB1 | Rabbit | Affinity (Golden, Colorado, USA) | AF5161 | 1:1000 |
| Total TOR | Rabbit | Affinity (Golden, Colorado, USA) | AF6308 | 1:1000 |
| p-TOR Ser 2448 | Rabbit | Affinity (Golden, Colorado, USA) | AF3308 | 1:1000 |
| anti-NF-κB p65 | Rabbit | Affinity (Golden, Colorado, USA) | AF5006 | 1:750 |
